# Stage-Specific Effects of Climatic Variation on Rice Yield and Nitrogen Use Efficiency for Identifying Adaptive Genotypes Based on Variable Selection Method

**DOI:** 10.3390/plants15040639

**Published:** 2026-02-18

**Authors:** Yingjun Ma, Xianglong Liang, Zhongqi Li, Pulin Kong, Huimin Zhang, Jinxia Xiang, Zhiyao Tian, Mingniang Qi, Ziyang Qu, Xianyang Li, Biqi Lei, Chanakan Prom-u-thai, Xiaorong Fan

**Affiliations:** 1 Sanya Institute of Nanjing Agricultural University, Sanya 572024, China; 2020203032@stu.njau.edu.cn (Y.M.); 2023103099@stu.njau.edu.cn (Z.L.); 2022103114@stu.njau.edu.cn (H.Z.); 2019203051@njau.edu.cn (J.X.); 9211310606@stu.njau.edu.cn (Z.T.); 2024103094@stu.njau.edu.cn (M.Q.); qzyang@njau.edu.cn (Z.Q.); 2022203019@stu.njau.edu.cn (X.L.); biqilei@stu.njau.edu.cn (B.L.); 2 State Key Laboratory of Crop Genetics & Germplasm Enhancement and Utilization, College of Resources and Environmental Sciences, Nanjing Agricultural University, Nanjing 210095, China; 3 Zhongshan Biological Breeding Laboratory, Nanjing 210095, China; 4 Department of Statistics and Data Science, Southern University of Science and Technology, 1088 Xueyuan Avenue, Shenzhen 518055, China; xianglongliang@gmail.com; 5 Institute of Grain and Oil, Meizhou Academy of Agriculture and Forestry Sciences, Meizhou 514000, China; rhythmsaber8@gmail.com; 6 Lanna Rice Research Center, Chiang Mai University, Chiang Mai 50200, Thailand; chanakan.p@cmu.ac.th; 7 Department of Plant and Soil Sciences, Faculty of Agriculture, Chiang Mai University, Chiang Mai 50200, Thailand

**Keywords:** climatic variation, rice, yield, nitrogen use efficiency, LASSO regression, ecological adaptability, nitrogen metabolic enzymes

## Abstract

Extreme weather variability across different climatic regions severely threatens rice yield and nitrogen use efficiency (NUE). To clarify the response of rice traits to climatic factors and optimize adaptive strategies, this study conducted field experiments in Nanjing (subtropical monsoon climate, 2022–2024) and Sanya (tropical marine climate, 2024). Nine rice genotypes covering indica, japonica, and hybrid genetic backgrounds were used, and the least absolute shrinkage and selection operator (LASSO) regression model was applied to identify the key drivers among climatic factors (air temperature, solar radiation, rainfall) and nitrogen application rates. Results showed growth stage-specific responses of rice to climatic stress: high-temperature stress during the flowering stage and low-temperature stress during the filling stage were key yield-limiting factors, while rainfall during the seedling stage and solar radiation during the tillering stage positively promoted yield and NUE. Nitrogen metabolic enzyme activities during the filling stage were the core physiological link connecting environmental stress and yield (R^2^ = 0.776–0.795). Furthermore, three genotypes (YZ2, IR30, and YZ3) were observed to show more positive associations with yield and NUE. This study clarifies the differential associative effects of climatic factors in tested environments and seasons, providing theoretical support and genetic resources for rice adaptive improvement under diverse climatic conditions.

## 1. Introduction

Climate change is one of the most pressing environmental challenges facing the world today. The Blue Book shows that China’s average annual surface temperature has continued to rise. Under these climatic conditions, rice frequently encounters high-temperature weather during the flowering stage [1,2]. The flowering period of rice is most sensitive to heat stress, as high temperatures reduce pollen viability, leading to a decline in grain yield of up to 10.5% [3,4].

Nitrogen topdressing before the flowering stage is a core strategy for mitigating high-temperature stress during anthesis, as it specifically counteracts the damage caused by heat to photosynthesis and reproductive development. Applying 25 kg/ha of nitrogen fertilizer 1.5 weeks before heading can increase the yield of rice genotypes with different heat tolerances by 16.9% to 23.7% [5]. However, crop NUE from soil is only 30–50%, and high temperatures can further reduce NUE [6]. Nitrogen loss leads to severe environmental pollution, and excessive nitrogen fertilizer application can cause a series of problems, including groundwater contamination [7]. Furthermore, excessive nitrogen fertilizer use contributes to increased greenhouse gas emissions, ultimately exacerbating global warming [8,9].

Nitrogen metabolism enzymes, namely, Nitrate Reductase (NR) and Glutamine Synthetase (GS), are core functional regulators of nitrogen uptake, assimilation, and translocation in rice. Changes in their activity levels directly influence rice NUE [10,11]. Moreover, the activities of NR and GS are temperature-sensitive. Low temperatures significantly decrease Glutamine Synthetase Activity (GSA) and Nitrate Reductase Activity (NRA) [12], thereby inhibiting nitrogen assimilation [13,14]. High temperatures similarly suppress the activity of these nitrogen metabolism enzymes [15].

Photosynthesis, as the foundation of carbon metabolism, works synergistically with nitrogen metabolism to regulate crop yield formation [16,17]. Both high and low temperatures can inhibit photosynthetic carbon dioxide fixation through various pathways, such as suppressing chlorophyll synthesis, reducing the products of the light-dependent reactions, and decreasing intercellular carbon dioxide concentration [18,19].

Although existing studies have clarified the response of rice traits to single environmental stresses, there are still obvious limitations in current research: (1) most data come from controlled greenhouse experiments, lacking systematic field observations across contrasting climates to reflect real-world complex climatic impacts; (2) correlation analysis mostly relies on simple correlation tests, failing to effectively quantify the independent regulatory contributions of environmental stresses and variety genetic backgrounds to yield and NUE.

To bridge these gaps, we conducted multi-environment field experiments across two contrasting climatic regions (Nanjing, subtropical monsoon; Sanya, tropical marine) from 2022 to 2024, and employed a variable selection method (LASSO regression). This design enables us to quantify the independent and stage-specific impacts of climatic factors, uncover their physiological linkage via nitrogen metabolism enzymes, and pinpoint adaptive genetic resources. Drawing upon previous breeding work, nine rice genotypes covering indica, japonica, and hybrid genetic backgrounds were used as test subjects. The large latitudinal span between the two locations creates natural heterogeneity in key climatic factors such as air temperature and solar radiation. Furthermore, as japonica rice is traditionally cultivated in Nanjing and indica rice is commonly grown in Hainan, the experimental setup aligns closely with local agricultural practices.

This study systematically determined climatic factors, nitrogen metabolic enzyme activities, net photosynthetic rate (NPR), yield, and NUE across different ecological regions and growth stages. To accurately quantify the independent effects of climatic factors and nitrogen application while avoiding multicollinearity interference, this study employed the LASSO regression model: this model introduces L_1_ regularization into traditional linear regression, controls regularization strength via a tuning parameter, imposes penalties on the absolute values of regression coefficients, and can shrink the coefficients of predictors unrelated to the response variable to zero, thereby enabling efficient variable selection [20]. Combined with this model, this study aims to achieve three objectives: (1) to quantify the independent contributions of climatic factors and nitrogen application to rice yield and NUE; (2) to elucidate the central role of nitrogen metabolic enzyme activities during the filling stage in linking environmental stress to yield; (3) to identify candidate genotypes showing favorable responses under climatic variability across contrasting agro-ecological regions.

## 2. Results

### 2.1. Air Temperature and Rainfall Conditions

**Figure 1:**
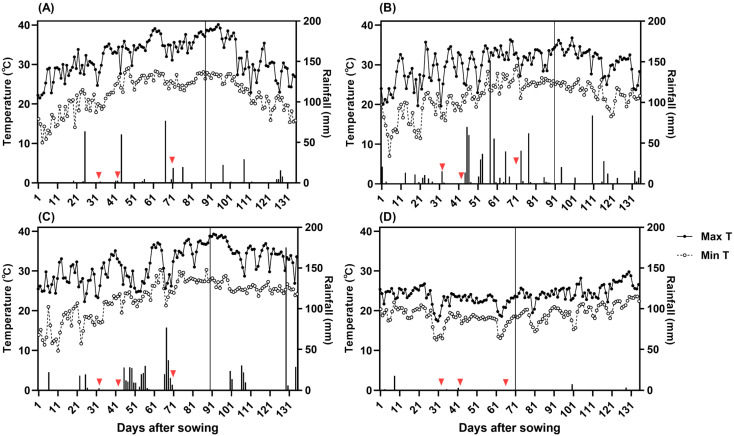
Air temperature and rainfall during the entire rice growth period under natural conditions from 2022 to 2024. The vertical solid black lines in the figure indicate the average time of heading initiation. Triangles indicate fertilizer application times. (**A**): 2022 Nanjing; (**B**): 2023 Nanjing; (**C**): 2024 Nanjing; (**D**): 2024 Sanya. Max T: Maximum temperature; Min T: Minimum temperature.

Field phenotypic observations were conducted on nine rice genotypes across two experimental sites. Air temperature data revealed substantial variations between sites and years. The 2023 Nanjing growing season was relatively mild, with an average of 18.7 days of high-temperature stress across the entire growth period. In contrast, the 2022 Nanjing and 2024 Nanjing seasons experienced more frequent high-temperature stress, with 43.4 and 38.7 days, respectively. The 2024 Sanya season was characterized by lower air temperatures, with no high-temperature stress events recorded but an average of 42.1 days of low-temperature stress throughout the growth cycle (Figure 1). All temperature stress days were calculated based on the threshold definitions detailed in Section 4.5, and the specific counts for each rice genotype are reported in the Supplementary Material Rar1.

Regarding rainfall, the 2022 Nanjing and 2024 Sanya seasons received less rainfall, while the 2023 Nanjing and 2024 Nanjing seasons received more. Notably, rainfall in the 2023 Nanjing season was concentrated primarily during the filling stage, whereas in the 2024 Nanjing season, it was concentrated during the tillering stage. In terms of solar radiation and sunshine duration, values recorded over the three years in Nanjing were consistently higher than those in Sanya (Figure S1).

### 2.2. Agronomic and Physiological Traits Under Different Climatic Conditions

**Figure 2:**
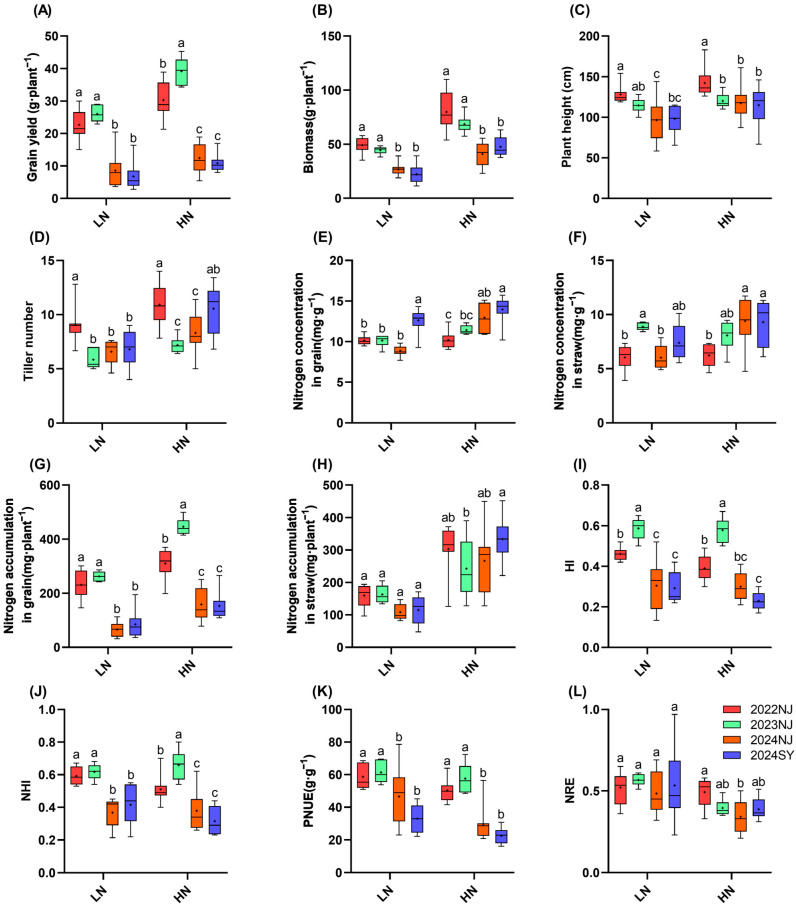
Agronomic traits and NUE of nine rice genotypes. (**A**): Grain yield; (**B**): Biomass; (**C**): Plant height; (**D**): Tiller number; (**E**): Nitrogen concentration in grain; (**F**): Nitrogen concentration in straw; (**G**): Nitrogen accumulation in grain; (**H**): Nitrogen accumulation in straw; (**I**): Harvest index; (**J**): Nitrogen harvest index; (**K**): Physiological nitrogen use efficiency; (**L**): Nitrogen recovery efficiency. All measurements were performed on a dry weight basis. LN: 75 kg·ha^−1^, HN: 250 kg·ha^−1^. *n* = 9 biological replicates; different lowercase letters indicate significant differences within the same nitrogen level (*p* < 0.05; Duncan’s multiple range test). The “+” symbol within each boxplot represents the mean value.

The results presented in Figure 2 demonstrate that grain yield varied significantly under different climatic conditions documented in Figure 1. The highest yield was observed in the 2023 Nanjing season, which experienced the fewest days of temperature stress, while seasons with more frequent high-temperature (2022 and 2024 Nanjing) or low-temperature stress (2024 Sanya) showed markedly reduced yields. Under the low-nitrogen condition, yields in the 2024 Nanjing and 2024 Sanya seasons were significantly lower than in the 2023 Nanjing season, decreasing by 68.3% and 73.9%, respectively (*p* < 0.05, Duncan’s multiple range test). Yield in the 2022 Nanjing season decreased by 12.9%, but this difference was not statistically significant. Under the high-nitrogen condition, yield in the 2023 Nanjing season was significantly higher than in all other season–location combinations. Compared to the 2023 Nanjing season, yields in the 2022 Nanjing, 2024 Nanjing, and 2024 Sanya seasons were reduced by 22.9%, 68.2%, and 72.3%, respectively.

Regarding plant height and tiller number, values were highest in the 2022 Nanjing season compared to other years and locations. Grain nitrogen concentration was highest in the 2024 Sanya season. For biomass, grain nitrogen accumulation, harvest index (HI), nitrogen harvest index (NHI), physiological nitrogen use efficiency (PNUE), and nitrogen recovery efficiency (NRE), a consistent pattern was observed under both low-nitrogen and high-nitrogen conditions: values were higher in the 2022 Nanjing and 2023 Nanjing seasons than in the 2024 Nanjing and 2024 Sanya seasons. Overall, increased nitrogen application raised nitrogen accumulation in both grains and straw but resulted in a decrease in NUE.

**Figure 3:**
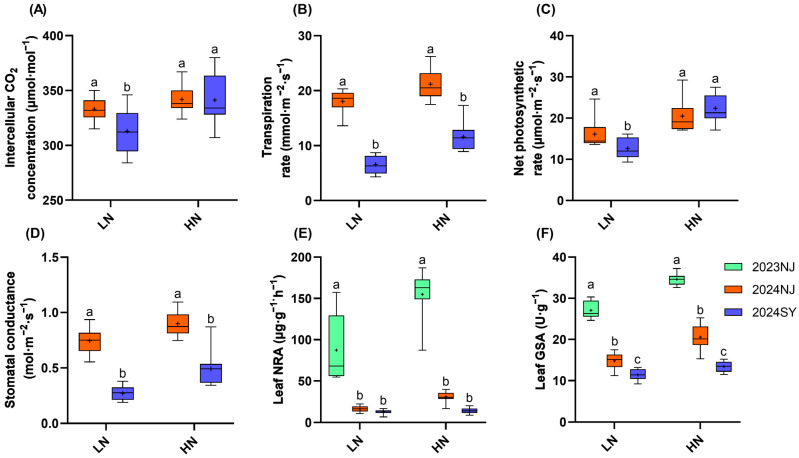
Photosynthetic and nitrogen metabolism enzyme activity traits during the filling stage of nine rice genotypes. (**A**): Intercellular carbon dioxide concentration during the filling stage; (**B**): Transpiration rate during the filling stage; (**C**): Net photosynthetic rate during the filling stage; (**D**): Stomatal conductance during the filling stage; (**E**): Leaf nitrate reductase activity during the filling stage; (**F**): Leaf glutamine synthetase activity during the filling stage. All enzyme activity units are based on fresh weight. LN: 75 kg·ha^−1^, HN: 250 kg·ha^−1^. *n* = 9 biological replicates; different lowercase letters indicate significant differences within the same nitrogen level (*p* < 0.05; Duncan’s multiple range test). The “+” symbol within each boxplot represents the mean value.

**Figure 4:**
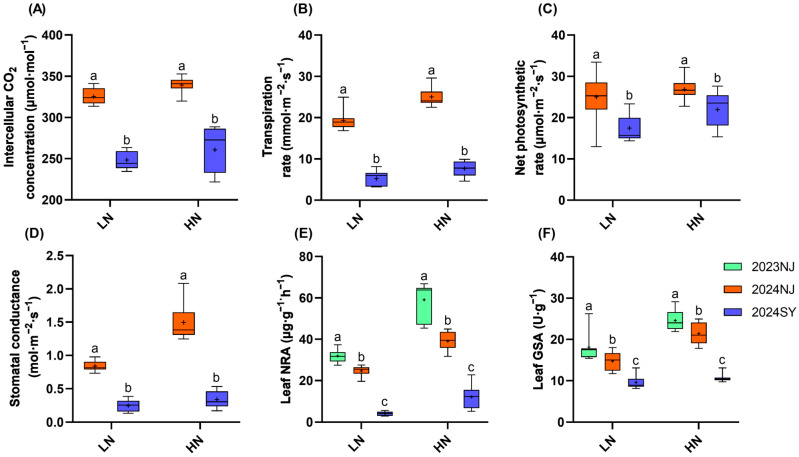
Photosynthetic and nitrogen metabolism enzyme activity traits at the booting stage of nine rice genotypes. (**A**): Intercellular carbon dioxide concentration during the booting stage; (**B**): Transpiration rate during the booting stage; (**C**): Net photosynthetic rate during the booting stage; (**D**): Stomatal conductance during the booting stage; (**E**): Leaf nitrate reductase activity during the booting stage; (**F**): Leaf glutamine synthetase activity during the booting stage. All enzyme activity units are based on fresh weight. LN: 75 kg·ha^−1^, HN: 250 kg·ha^−1^. *n* = 9 biological replicates; different lowercase letters indicate significant differences within the same nitrogen level (*p* < 0.05; Duncan’s multiple range test). The “+” symbol within each boxplot represents the mean value.

The results presented in Figure 3 indicate that both NRA and GSA in the 2023 Nanjing season were significantly higher than those in the 2024 Nanjing and 2024 Sanya seasons (*p* < 0.05, Duncan’s multiple range test). Specifically, for NRA under low-nitrogen treatment, the values in 2024 Nanjing and 2024 Sanya decreased by 81.4% and 85.5%, respectively, compared with 2023 Nanjing; under high-nitrogen treatment, the corresponding decreases reached 80.0% and 90.9%. As for GSA, under low-nitrogen treatment, its levels in 2024 Nanjing and 2024 Sanya were 45.4% and 58.8% lower than that in 2023 Nanjing, while under high-nitrogen treatment, the reductions were 59.3% and 61.2%, respectively. Furthermore, the 2024 Nanjing season exhibited higher values than the 2024 Sanya season for NRA, GSA, intercellular CO_2_ concentration, transpiration rate, stomatal conductance, and NPR. Increased nitrogen application enhanced both nitrogen metabolism enzyme activities and photosynthetic parameters. The trends for nitrogen metabolism enzyme activities and photosynthesis during the booting stage were consistent with those observed during the filling stage (Figure 4).

### 2.3. Correlation Analysis

**Figure 5:**
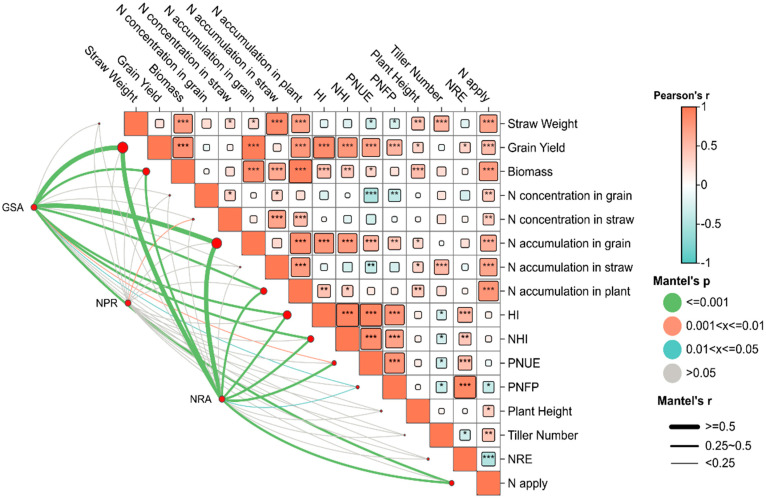
Correlation analysis of yield, NUE, enzyme activities, and NPR. The heatmap shows the Pearson correlation coefficients of the selected traits, where the depth of red indicates the strength of positive correlation and the depth of blue indicates the strength of negative correlation. *, **, *** indicate significance at *p* = 0.05, 0.01 and 0.001, respectively. The colors correspond to different ranges of Mantel test *p*-values: green represents *p* ≤ 0.001, orange represents 0.001 < *p* ≤ 0.01, blue represents 0.01 < *p* ≤ 0.05, and gray represents *p* > 0.05. The network diagram below the heatmap highlights the associations among traits, and the thickness of the lines corresponds to the magnitude of the Mantel correlation coefficient r: thin lines indicate r < 0.25, medium-thickness lines indicate 0.25 ≤ r < 0.5, and thick lines indicate r ≥ 0.5.

The Mantel test is a non-parametric statistical method used to analyze the correlation between two matrices. Its core function is to quantify the correlation strength between a multivariate dataset matrix and another multivariate dataset matrix, and it is particularly suitable for complex data systems involving multiple environments and traits [21]. In Figure 5, we integrated NRA, GSA, and NPR from the jointing and filling stages to construct a comprehensive physiological indicator matrix, which was then subjected to correlation analysis with traits such as yield and NUE. The core purpose is to quantify the overall correlation strength of nitrogen metabolic enzyme activities and NPR with yield formation. The results in Figure 5 show that both GSA and NRA exhibited highly significant positive correlations (*p* < 0.001) with grain yield, biomass, nitrogen accumulation in grain, nitrogen accumulation in plant, harvest index, and NHI. In contrast, NPR was only significantly correlated with nitrogen accumulation in straw (0.001 < *p* < 0.01). Additionally, Table S1 analyzes the correlation between each physiological indicator and yield separately by growth stage. The correlation between enzyme activities during the filling stage and yield (R^2^ > 0.75) was significantly higher than that during the booting stage (Table S1).

### 2.4. LASSO Regression Analysis of Rice Traits

**Figure A1:**
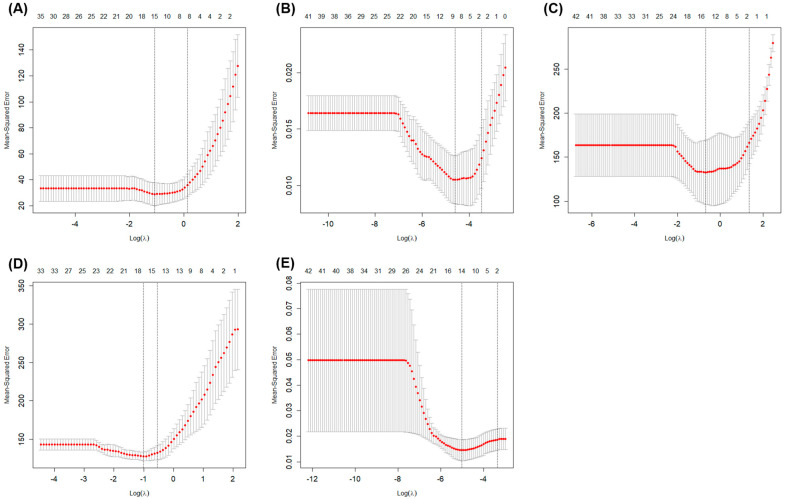
Changes in fitting deviation of grain yield and nitrogen use efficiency with different *λ* values. (**A**): Grain yield; (**B**): Nitrogen harvest index; (**C**): Physiological nitrogen use efficiency; (**D**): Nitrogen partial factor productivity; (**E**): Nitrogen recovery efficiency. The left vertical dotted line represents lambda.min and the right vertical dotted line represents lambda.1se. The red dots represent the mean-squared error, and the error bars represent the standard deviation of the mean-squared error.

In our study, 3-fold cross-validation was employed to select the tuning parameter *λ*, with the mean-squared error calculated therefrom. We adopted ‘lambda.min’, which minimizes the mean cross-validated prediction error, rather than ‘lambda.1se’, which yields a more parsimonious model corresponding to a more strongly regularized solution and may exclude predictors with moderate but meaningful effects. Given that the primary objective of this study was to maximize predictive performance and to fully capture the effects of variables on the response variables, ‘lambda.min’ was considered more appropriate. As illustrated in Figure A1 and Figure S2, the red dashed line denotes the MSE, and the error bars represent the standard deviation of the MSE. Two specific values within the *λ* sequence are marked by vertical dotted lines: lambda.1se and lambda.min. The lambda.min corresponds to the *λ* value that minimizes the mean cross-validation error. All results presented in this study were derived using lambda.min.

Additionally, LASSO estimates the effect coefficients of the selected variables [22]. Variables with larger effect coefficients contribute more to explaining the dependent variable; thus, these coefficients were adopted as the importance scores for the LASSO-selected variables. The results of LASSO regression are summarized in Table 1 and Figure A1. Negative effect coefficients (−β) signify a negative impact on the response variable, whereas positive coefficients (+β) indicate a positive impact.

The analysis based on Table 1 shows that environmental and varietal factors had a high explanatory power for grain yield (R^2^ > 0.8). Grain yield was positively correlated with nitrogen application, solar radiation during the tillering stage, rainfall during the seedling stage, and genotypes 4, 5, and 6. It was negatively correlated with the number of high-temperature stress days during the flowering stage, the number of low-temperature stress days during the filling stage, sunshine duration during the seedling stage, and rainfall during the booting, flowering, and filling stages. To provide context for the genotype-specific analyses, the mean yield of the nine rice genotypes across all tested environments is shown in Supplementary Figure S3.

The explanatory power of environmental and varietal factors differed for various NUE components. The nitrogen harvest index was positively correlated with solar radiation during the tillering stage, rainfall during the seedling stage, and variety 2, but negatively correlated with solar radiation during the filling stage, sunshine duration during the seedling stage, and genotypes 3, 8, and 9. PNUE was well explained (R^2^ = 0.808) and showed positive correlations with solar radiation during the tillering stage, rainfall during the seedling stage, and genotypes 2, 4, 5, and 6. It was negatively correlated with nitrogen application rate, the number of high-temperature stress days during the tillering stage, sunshine duration during the seedling stage, and rainfall during the flowering and filling stages.

Nitrogen partial factor productivity was positively correlated with rainfall during the seedling stage and genotypes 2, 5, and 6. It was negatively correlated with nitrogen application rate; the number of high-temperature stress days during the tillering and booting stages; the number of low-temperature stress days during the flowering and filling stages; solar radiation during the booting and filling stages; sunshine duration during the seedling and flowering stages; rainfall during the filling stage; and genotypes 1, 3, 7, and 8. The factors influencing NRE were similar to those for yield, but the model’s explanatory power was lower, with R^2^ of only 0.535.

**Figure 6:**
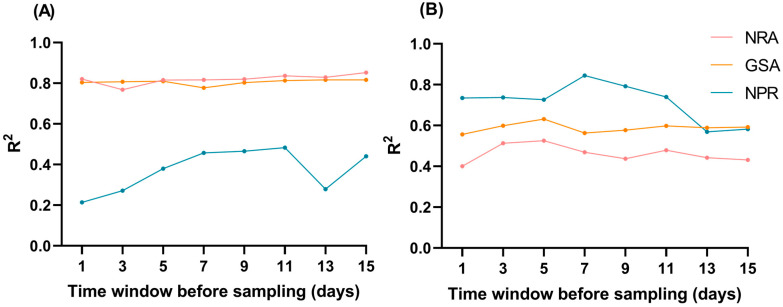
Optimal time window analysis of climatic factors affecting NRA, GSA and NPR. (**A**): Booting stage; (**B**): Filling stage. The X-axis represents the number of days in the time window before sampling, and the Y-axis represents the R^2^ value of the LASSO regression model. A higher R^2^ indicates a stronger explanatory power of climatic factors in the corresponding time window for the target physiological traits.

Nitrogen metabolism enzyme activities and NPR, as upstream physiological processes, are more susceptible to recent climate fluctuations than downstream traits such as yield. Ma et al. [23] found that 1–3 days of cold stress treatment (19 °C) resulted in significant fluctuations in enzyme activities and NPR, while 7 days of cold stress treatment caused a 26.73–82.52% decrease in nitrogen metabolism enzyme activities and a 39.94–46.21% decrease in NPR of functional leaves during the filling stage. To accurately quantify the effects of climatic factors on physiological traits and avoid interference from long-term cumulative effects, we conducted a time window analysis. We calculated the average values of each climatic factor within different day windows before sampling, specifically testing a series of time windows including 1–15 days. Subsequently, LASSO regression models were established by taking the climatic data calculated for each window as independent variables and the nitrogen metabolism enzyme activities and NPR during the booting and filling stages as dependent variables. By comparing the R^2^ of the models across different windows, the window with the highest R^2^ was identified as the optimal time window for the climatic factor to affect the target physiological trait. The time window analysis showed that the key climatic period affecting nitrogen metabolism enzyme activities during the filling stage was seven days before sampling. The LASSO model established using the climatic data calculated from this window exhibited the highest explanatory power for the variation in enzyme activities (Figure 6).

Table 2 presents the LASSO regression results of nitrogen metabolism enzyme activities and NPR with the average climatic data seven days before sampling. In general, both nitrogen metabolism enzyme activities and NPR were positively associated with nitrogen application rate. For nitrogen metabolism enzyme activities, both rainfall and maximum air temperature exhibited significant positive contributions. Additionally, nitrogen metabolism enzyme activities at the booting stage were positively correlated with minimum air temperature. NPR was positively correlated with sunshine duration across all stages. Among them, NPR at the filling stage had a higher explanatory power, reaching 0.845, and was correlated with eight genotypes. Among the climatic factors, both solar radiation and rainfall showed positive correlations. In contrast, NPR at the booting stage had a lower explanatory power of only 0.457, and was positively correlated with both minimum and maximum air temperature.

## 3. Discussion

### 3.1. Nitrogen Fertilizer Mitigates the Negative Effects of Temperature Stress on Yield, NUE, and Enzyme Activities

High-temperature stress is a key challenge in rice cultivation. Existing research has confirmed that rising temperatures significantly negatively impact rice yield and NUE [24]; similarly, low temperatures significantly reduce rice seed setting rate, consequently leading to yield decline [25]. For instance, Zhu et al. [26] found that increased nitrate supply under high-temperature conditions can induce the nuclear localization of the *NLP3* gene in rice, thereby activating the expression of heat-tolerance-related genes such as *HsfA3*/*A7*, ultimately enhancing plant thermotolerance. This provides a molecular-level reference for understanding reported temperature stress responses in rice.

This study further indicates that both high- and low-temperature stresses significantly reduce rice yield and NUE, with the flowering stage being particularly sensitive to extreme temperatures: both high- and low-temperature stress during this period significantly inhibit yield formation and NUE improvement. Specifically, based on the LASSO regression analysis results (Table 1), yield showed significant negative correlations with the number of high-temperature stress days during the flowering stage (β = −0.164) and the number of low-temperature stress days during the filling stage (β = −0.686), consistent with the conclusions of our team’s previous research [6]. The physiological responses associated with these two types of stress appear to differ: high temperature during flowering directly damages pollen viability, leading to a significant decrease in the seed-setting rate and ultimately causing a sharp yield reduction. In contrast, low temperature during the filling stage is associated with greater yield loss, potentially related to two factors: on one hand, it is linked to reduced root nitrogen uptake capacity, and on the other hand, it may correlate with impaired plant nitrogen assimilation. Together, these effects slow grain-filling rate and reduce dry matter accumulation efficiency [24,27].

From the perspective of nitrogen fertilizer regulation effects, this study found that increasing nitrogen application could significantly increase the nitrogen concentration and accumulation in grains and straw (Figure 2, Table S2). Meanwhile, it alleviated partial climatic stress by enhancing nitrogen metabolism enzyme activities (Table 2; nitrogen application rate showed a positive correlation with all enzyme activities) and simultaneously improved NPR. However, on the other hand, increasing nitrogen application also resulted in a significant decrease in NUE. This result is consistent with previous research findings: increasing nitrogen application can mitigate stress by promoting photosynthesis and nitrogen metabolism, but excessive nitrogen input will reduce NUE [6,28,29]; under different temperature conditions, increasing nitrogen application can further increase grain protein content [30]. Therefore, optimizing nitrogen application rate represents an important management strategy for balancing rice performance under climatic variability and NUE, but further research is needed to determine the specific optimal nitrogen application rate. In future rice cultivation and management, it is necessary to establish a dynamic balance between “nitrogen fertilizer alleviating climatic stress” and “NUE improvement”, rather than merely relying on increasing nitrogen application to cope with extreme climates.

### 3.2. Sunshine Duration and Solar Radiation Are Key Factors Constraining Rice Yield

As the core driver of photosynthetic metabolism and dry matter accumulation in rice, the spatiotemporal heterogeneity of solar radiation plays a significantly complex role in regulating rice yield formation and NUE. Existing studies indicate a close association between solar radiation and rice yield. For example, Zhou et al. [31] found that the correlation coefficient between the yield of medium-maturing japonica rice and total solar radiation across the entire growth period reached 0.884, and varietal maturity significantly influenced the strength of this association (e.g., the correlation coefficient for late-maturing japonica rice was only 0.714). Simultaneously, an interactive effect exists between solar radiation and nitrogen; studies on lettuce have observed their synergistic regulation of crop yield and photosynthetic rate [32]. This suggests that the effects of the solar radiation require comprehensive analysis in conjunction with crop species, varietal characteristics, and cultivation conditions.

This study further identified growth stage-dependent association patterns between solar radiation factors and rice traits through LASSO regression analysis (Table 1): Solar radiation during the tillering stage exhibited a significant positive correlation with yield (β = 0.949), NHI (β = 0.00125), PNUE (β = 0.716), and NRE (β = 0.0116); in contrast, sunshine duration during the seedling and flowering stages and solar radiation during the booting and filling stages showed significant negative correlations with the aforementioned traits. It is noteworthy that several shade-related studies have reported that shading treatments (blocking 30–80% of solar radiation) significantly reduce rice yield, nitrogen metabolism enzyme activities, and NUE [33,34,35], which differs from the conclusions of this study. The core reasons can be explained from two aspects:

Firstly, there is a general “inverted U-shaped” non-linear relationship between solar radiation and total rice yield [36]: when solar radiation is below the critical threshold, increasing sunshine duration can significantly promote the accumulation of photosynthates, thereby improving yield; the core characteristic of shading experiments is “extreme low-light stress”, which far exceeds the crop’s solar radiation compensation point and exerts a unidirectional inhibitory effect on physiological metabolism; when solar radiation intensity exceeds the critical threshold, a continuous increase may be associated with photoinhibition or energy waste, which correlates with a subsequent decrease in yield. Moreover, this threshold varies with regional climate and the solar radiation response characteristics of genotypes. Cai [37] also confirmed that in the middle and lower reaches of the Yangtze River, the comprehensive impact intensity of photosynthetically active radiation on the yield of early rice and single-cropping rice is significantly higher than temperature and precipitation, and it indirectly reduces yield by inhibiting net primary productivity; when photosynthetically active radiation exceeds the utilization capacity of the rice photosynthetic system, excess energy is released in the form of heat dissipation, resulting in a decrease in CO_2_ assimilation rate and reduced accumulation of net primary productivity; at the same time, high nighttime temperatures in this region enhance respiration, which additionally consumes photosynthates and further amplifies the inhibitory effect of strong solar radiation on yield. This synergistic effect is particularly prominent during the growing seasons of early rice and single-cropping rice [37,38,39].

Secondly, the planting density design in this study (one seedling per hill, plant and row spacing of 20 cm) exerted a key regulatory effect on solar radiation factors: in conventional rice production, the density design of two to four seedlings per hill aims to improve canopy solar radiation capture efficiency through population advantages [3,30], while the lower planting density in this experiment allowed individual plants to obtain far more solar radiation resources than needed, making it easier to reach light saturation. This is also one of the important reasons for the more significant negative effects of solar radiation in this study [37].

From the perspective of physiological mechanisms, the tillering stage is a critical vegetative phase for rice to construct carbon skeletons and accumulate photosynthates. During this period, sufficient and stable solar radiation can promote chlorophyll synthesis and photosynthetic electron transport efficiency, providing a carbon source for yield formation [40], which also explains the positive regulatory effect of sunshine duration during the tillering stage on yield and NUE-related indicators. However, from the booting stage to the filling stage, there were varying degrees of temperature stress in all environments of this experiment (35.8 days of high-temperature stress in 2022 Nanjing, 33.3 days of high-temperature stress in 2024 Nanjing, and 23.4 days of low-temperature stress in 2024 Sanya). Previous studies have reported that drastic temperature changes are often accompanied by rapid fluctuations in solar radiation, which can disrupt the coordination mechanism between leaf light capture and light reactions, and exacerbate oxidative stress damage [41].

It should be noted that this study was only conducted at two experimental sites (Nanjing and Sanya), and the applicability of the above conclusions regarding the effects of solar radiation factors on rice traits is limited. Their generalizability needs to be further verified in more climatic zones. This study demonstrates that LASSO regression can identify the environmental factors restricting crop traits under multiple environments. In the two experimental sites, the spatiotemporal matching of solar radiation and sunshine duration represents one of the dominant limiting associations affecting rice yield and NUE within the tested environments. In cultivation, radiation use efficiency can be improved through variety improvement (enhancing photoinhibition resistance) and optimization of field management (such as reasonably dense planting to adjust canopy solar radiation distribution), thereby alleviating strong solar radiation stress. This also provides a technical reference for subsequent multi-environment crop climate adaptability research.

### 3.3. Rainfall During the Seeding Stage Is Crucial for Nitrogen Metabolism Enzyme Activity in Rice

This study found that rainfall was significantly correlated with rice yield, NUE, and nitrogen metabolism enzyme activities (Table 1, Table 2 and Table S2), and this correlation exhibited clear growth stage-specificity: rainfall during the seedling stage showed a significant positive correlation with yield, NHI, PNUE, and NPFP (Table 1). However, rainfall during the booting stage, flowering stage, and filling stage was significantly negatively correlated with yield and NUE. This may be due to the fact that rainfall can alter nitrogen availability, and excessive rainfall can lead to nitrogen loss [42]. As a core environmental factor regulating rice growth and development, the spatiotemporal distribution heterogeneity of rainfall exerts a critical regulatory role on rice yield formation and NUE by influencing water supply, soil nutrient migration efficiency, and plant physiological metabolic processes [43]. Previous studies have confirmed that appropriate rainfall during the tillering stage and booting stage can improve yield by optimizing water and nutrient availability, and increased rainfall during the vegetative growth stage can significantly enhance the plant stem-to-grain ratio [44]. Mechanistically, heavy rainfall events act as a critical hydrological driver that can exacerbate nitrogen removal from paddy systems. Intense precipitation can trigger substantial hydrological transport of soil nutrients [45]. In addition, this study included genotypes with three genetic backgrounds: indica rice, japonica rice, and hybrid rice. Different genotypes have inherent differences in root characteristics and waterlogging tolerance [44]. Whether this differentiation in genetic background will amplify the negative effects of rainfall during the reproductive growth stage remains unclear and requires further research.

### 3.4. The Critical Role of Nitrogen Metabolism Enzyme Activities During the Filling Stage in Yield Formation

This study further identified strong associations between nitrogen metabolism enzyme activities and yield-related traits under the tested climatic conditions: first, both GSA and NRA showed extremely significant positive correlations with yield, biomass, grain nitrogen accumulation, HI, and NHI (Figure 5). Moreover, the explanatory power of enzyme activities during the filling stage for yield (R^2^ > 0.75) was significantly higher than that of nitrogen metabolism enzyme activities during the booting stage and NPR during the filling stage (Table S1), suggesting that variation in nitrogen metabolism enzyme activities showed a stronger statistical association with yield differences than NPR. Consistent with previous studies, nitrogen metabolism enzymes are widely recognized as key components involved in nitrogen absorption, assimilation, and translocation processes in rice. Among them, GS and NR participate in major pathways of nitrogen assimilation: GS is responsible for assimilating ammonium nitrogen absorbed by roots and recycled ammonium nitrogen in plants into amino acids, serving as the core of nitrogen translocation from vegetative organs to grains [46]; NR, as a rate-limiting enzyme in nitrogen assimilation, can catalyze the reduction of nitrate in soil to nitrite, providing a basic nitrogen source for subsequent amino acid and protein synthesis [47]. Previous studies have confirmed that overexpression of related genes such as *OsGS1;2* and *OsNR2* can significantly enhance the nitrogen absorption and accumulation capacity of rice [10,47,48]; increasing nitrogen application can also improve yield by enhancing nitrogen metabolism enzyme activities [49]. Notably, temperature stress can significantly inhibit GSA and NRA during the filling stage, hindering the directional translocation of nitrogen to grains and ultimately leading to crop yield reduction [50,51], which provides supporting evidence for understanding the physiological associations underlying rice yield variation under climatic variability.

Previous related studies have mostly focused on a single environment (such as controlled greenhouse conditions) or a few specific materials, and the systematic correlation between nitrogen metabolism enzyme activities, yield, and NUE has not been clarified in a multi-environment and multi-variety system; this study fills this gap. Meanwhile, this study also confirmed that increasing nitrogen application can significantly enhance GSA and NRA, and climatic factors such as temperature and rainfall are positively correlated with GSA and NRA (Table 2). However, it should be noted that there is a lack of research on the temperature thresholds for nitrogen metabolism enzyme activities, so Table 2 does not further analyze the differential effects of high and low temperature stress on GSA and NRA; the time window analysis in Figure 6 shows that the climatic data 7 days before sampling has the highest explanatory power for nitrogen metabolism enzyme activities and NPR, which provides a clear research direction for subsequent in-depth exploration of the climatic thresholds of enzyme activities and photosynthetic characteristics.

### 3.5. LASSO Regression Reveals Genotype-Specific Response Patterns to Climatic Variation

Under the context of increasing climatic variability, rice genotypes exhibit significant genotypic differences in their responses to environment. This “genotype × environment interaction effect” plays an important role in shaping crop field phenotypes [52]. Developing rice materials with high yield and resource-use efficiency remains a central objective in modern breeding programs, particularly under increasing climatic variability. Through LASSO regression analysis (Table 1), this study found that genotypes 4, 5, and 6 showed significant positive correlations with yield and NUE, among which, genotype 6 was also significantly positively correlated with NRA during the filling stage and GSA during the booting stage (Table 2). These results indicate that under varying environmental conditions, genotype 6 exhibits a coordinated association between yield, NUE, and nitrogen metabolism enzyme activities, suggesting a coordinated response pattern within the tested environments that warrants further validation. However, there are few studies applying LASSO regression to germplasm screening to date. Thus, the biological significance of the strong correlations between genotype 6 and these traits, as well as their practical applications in actual production, require further investigation.

### 3.6. Methodological Advantages of LASSO Regression for Deciphering Field-Scale Climate–Trait Associations

The objective of this study was not to construct a universally predictive weather–yield equation, but rather to conduct an exploratory attribution analysis under realistic field conditions. Specifically, we aimed to identify dominant statistical association patterns among climatic variables, nitrogen application, and rice traits within the tested environments.

In recent agronomic research, particularly for high-dimensional datasets, LASSO regression has demonstrated practical value for extracting key predictors while controlling model complexity. For example, in hyperspectral identification of rice seed purity, LASSO reduced 176 spectral variables to 3–17 critical wavelengths while maintaining a classification accuracy above 98% [53]. Similarly, UAV-based multispectral analyses combined with LASSO enabled rapid and accurate prediction of spring wheat yield [54]. These applications illustrate how coefficient penalization and feature selection can enhance interpretability and reduce overfitting in complex biological datasets [55].

Despite these advances, identifying dominant climatic signals under field-scale variability remains analytically challenging. Traditional analyses of climate–trait relationships often rely on pairwise correlations or conventional linear models. Such approaches may struggle to disentangle the relative contributions of climatic predictors, thereby complicating the interpretation of individual weather effects. Comparative studies based on two-year field observations have identified temperature or solar radiation as major determinants of rice yield [56,57]; however, these analytical frameworks may not fully account for multicollinearity among meteorological factors. This may also contribute to inconsistencies among studies employing different statistical approaches [6,43]. In this context, LASSO regression provides an alternative perspective by prioritizing predictors that retain independent statistical associations with target traits despite environmental interdependence.

While LASSO is well suited for detecting dominant association structures in high-dimensional settings, the robustness and transferability of these patterns should be validated via additional field trials [20]. Therefore, the climatic signals identified here should be interpreted as field-scale statistical attributions rather than universal drivers, providing focused hypotheses for subsequent mechanistic validation and multi-location testing.

### 3.7. Limitations and Future Perspectives

Although this study systematically revealed the association patterns between climatic factors, nitrogen application rate, and rice traits, there are still three limitations: first, the inference of this study is constrained by the limited number of macro-environments. Although LASSO regression can identify dominant association patterns under high-dimensional conditions, the generality and predictive capacity of these associations must be validated using broader and independent multi-environment datasets; second, the determination of physiological indicators lacks complete temporal continuity, with non-anthropogenic data gaps including the absence of nitrogen metabolism enzyme activities in 2022 Nanjing and NPR in 2022 Nanjing and 2023 Nanjing. Notably, all physiological and agronomic traits are complete for the 2024 Nanjing and 2024 Sanya trials, which meets the data requirements for general field studies. These gaps may limit the comprehensiveness of interpretations across all site-year combinations; third, the potential interference effects of soil and field management were not quantified.

Future studies can be optimized targeting these limitations: expand the range of tested genotypes (including different subspecies such as indica rice, japonica rice, and AUS rice), add experimental locations with diverse climate types, improve the full-cycle dynamic monitoring of physiological indicators, and quantify the interference effects of soil and field management, so as to more comprehensively characterize rice responses to climatic variation.

## 4. Materials and Methods

### 4.1. Experimental Design

A total of nine rice genotypes, covering three genetic backgrounds (indica, japonica, and hybrid), were used in this study. These included three parental varieties and six elite breeding lines selected from the F_8_ generation. The parental materials comprised the nitrogen-efficient japonica variety DFE02 (with applied variety rights), and the nitrogen-efficient indica varieties KASALATH and IR30. The six selected lines (YZ1 to YZ6) were derived from crosses between DFE02 and either KASALATH or IR30, followed by eight generations of selfing and multi-trait directional selection targeting yield and nitrogen efficiency to obtain genetically stable genotypes (Table 3). Seeds were sown on 12 May 2022, 29 April 2023, and 5 May 2024, in the experimental field of Nanjing Agricultural University in Lishui District (31°35′ N, 119°10′ E), Nanjing, Jiangsu Province. Transplanting was carried out on 4 June 2022, 7 June 2023, and 13 June 2024, respectively. Before sowing, soil samples were collected, air-dried, and analyzed for physicochemical properties. The soil in Nanjing is characterized as a clay loam. The basic soil properties at the Nanjing site were pH 7.5, soil moisture content 3.3%, organic matter 6.9 g·kg^−1^, total nitrogen 0.7 g·kg^−1^, available potassium 100.2 mg·kg^−1^, and available phosphorus 8.0 mg·kg^−1^. Additionally, the genotypes were planted in Yazhou (18°15′ N, 109°30′ E), Sanya, Hainan Province, with sowing on 5 December 2023 and transplanting on 3 January 2024. The soil in Sanya is characterized as a clay loam. The basic soil properties in Sanya were pH 5.8, soil moisture content 3.0%, organic matter 8.2 g·kg^−1^, total nitrogen 0.7 g·kg^−1^, available potassium 97.6 mg·kg^−1^, and available phosphorus 15.1 mg·kg^−1^.

### 4.2. Fertilizer Application

The macronutrient fertilizers (nitrogen, phosphorus, and potassium) used in this experiment were urea (containing 46% N), single superphosphate (containing 16% P_2_O_5_), and potassium chloride (containing 60% K_2_O), respectively. The field management followed local conventional practices, including continuous flooding of rice fields, and pest, weed, and disease control consistent with standard local rice cultivation methods. One seedling was planted per hill, with a planting density of 70 seedlings per plot. Two nitrogen fertilizer gradients were designed in 2024 Nanjing and Sanya: low-nitrogen (LN) and high-nitrogen (HN), with application rates of 75 kg·ha^−1^ and 250 kg·ha^−1^ (calculated as pure nitrogen), respectively. Nitrogen treatments were also applied in the other years, with detailed information provided in Supplementary Materials. Nitrogen fertilizer was applied in three splits as base fertilizer, tillering fertilizer, and booting fertilizer at a ratio of 4:3:3. Phosphorus fertilizer (P_2_O_5_) and potassium fertilizer (K_2_O) were applied once as base fertilizers at rates of 75 kg·ha^−1^ and 166 kg·ha^−1^, respectively. The planting spacing was 20 cm between plants and 20 cm between rows. The experiment was arranged in a split-plot design, with nitrogen fertilizer treatments as the main plots and genotypes as the subplots. Each treatment was replicated three times, and the area of each plot was 2.8 m^2^ (2 m × 1.4 m).

### 4.3. Sample Collection

Samples at the booting stage were collected seven days before flowering, and samples at the filling stage were collected 20 days after flowering, both on sunny days. Ten rice plants were randomly selected from each plot (excluding border plants) to measure indicators such as plant height and tiller number. From these ten plants, five plants with traits close to the average values were selected and harvested at ground level. After harvesting, the selected plants were placed in an oven at 105 °C for 30 min to terminate metabolism, and then transferred to an oven at 70 °C to dry to a constant weight. After weighing each part of the sample, the plants were ground using a plant grinder. In this experiment, all plant samples consisted of above-ground parts only, excluding roots.

### 4.4. Sample Analysis and Measurement Methods

The determination of nitrogen content in rice plants was conducted as follows: harvested samples were heated at 105 °C for 30 min and then ground into powder. Total nitrogen content was determined using the H_2_SO_4_-H_2_O_2_ digestion method followed by continuous flow analysis with an autoanalyzer (CFAAA3, Bran & Luebbe Inc., Norderstedt, Germany). 

The calculation of NUE referred to the method of Zhang [58]:

(1) $$NHI=\frac{Nitrogen\;accumulation\;in\;grains\;per\;plant}{Total\;nitrogen\;accumulation\;in\;above\;ground\;plant\;per\;plant}$$

(2) $$NRE(g/g)=\frac{Total\;nitrogen\;accumulation\;in\;above\;ground\;plant\;per\;unit\;area}{Nitrogen\;application\;rate}$$

(3) $$NPFP(g/g)=\frac{Grain\;yield\;per\;unit\;area}{Nitrogen\;application\;rate}$$

(4) $$PNUE(g/g)=\frac{Grain\;yield\;per\;plant}{Total\;nitrogen\;accumulation\;in\;above\;ground\;plant\;per\;plant}$$

where NHI is the nitrogen harvest index, NRE is nitrogen recovery efficiency, NPFP is nitrogen partial factor productivity, PNUE is physiological nitrogen use efficiency, and nitrogen application rate is the nitrogen applied per unit area (calculated as pure nitrogen).

The NPR was measured using a Li-6800 photosynthesis system, with the following settings: light intensity of 1500 μmol·m^−2^·s^−1^, CO_2_ concentration of 400 μmol·mol^−1^, and air humidity of 50%. Measurements were performed after the instrument stabilized, between approximately 9:00 and 11:00 on windless and sunny mornings. For each treatment, three uniformly growing hills were selected, with three replicates. The penultimate leaf was measured at the booting stage, and the flag leaf was measured at the filling stage.

Sampling for nitrogen metabolism enzyme activity was conducted on the same morning as the photosynthesis measurement. Three uniformly growing plants were selected for each treatment, with three replicates. The penultimate leaf was collected at the booting stage, and the flag leaf was collected at the filling stage. NRA was measured by the sulfanilamide colorimetric method [59], and GSA was determined based on the ferric chloride colorimetric method [60]. For GSA, one unit (U) was defined as the amount of enzyme required to generate 1 μmol of γ-glutamylhydroxamate per hour per gram of fresh leaf tissue. For NRA, the activity was expressed as the mass of nitrite produced per hour per gram of fresh leaf tissue, with the unit of μg·g^−1^·h^−1^.

Measurements of climatic factors, grain yield, and NUE components were conducted across all site-year combinations (NJ2022, NJ2023, NJ2024, SY2024). Measurements of nitrogen metabolism enzyme activities (NRA, GSA) were performed in NJ2023, NJ2024, and SY2024, while photosynthetic parameters were determined in NJ2024 and SY2024.

### 4.5. LASSO Regression and Data Analysis

LASSO regressions were performed with R 4.5.2 (R Foundation for Statistical Computing, Vienna, Austria). The penalty parameter λ of LASSO regression was selected using the ‘glmnet’ package (4.1.0). Predictors (rice genotypes) were encoded as categorical variables (dummy-coded before model fitting; standardization applied) and predictors (climatic factors, nitrogen application) were encoded as continuous variables (standardization applied). The penalty parameter *λ* was selected via 3-fold cross-validation using lambda.min, to maximize predictive performance.

In ordinary linear regression (OLR), the parameter estimates are obtained by

(5) $${\hat{\beta }}^{OLR}=\underset{\beta }{\mathrm{argmin}}\sum_{i=1}^{n}{\left(y_{i}-x_{i}^{T}\beta \right)}^{2}.$$

In LASSO, we add a penalty to (5) and the parameter estimates of LASSO are obtained by

(6) $${\hat{\beta }}^{LASSO}=\left\{\underset{\beta }{\mathrm{argmin}}\sum_{i=1}^{n}{\left(y_{i}-x_{i}^{T}\beta \right)}^{2}+\lambda \sum_{j=1}^{p}\left|\beta_{j}\right|\right\}.$$

where $y_{i}$ denotes the $i^{th}$ response, $x_{i}={\left(x_{i1},\ldots ,x_{ip}\right)}^{T}$ is the vector of predictors, $\beta ={\left(\beta_{1},\ldots ,\beta_{p}\right)}^{T}$ are regression coefficients, *n* is the sample size, *p* is the number of predictors, and λ≥0 is a tuning parameter controlling the strength of shrinkage. The $l_{1}$ penalty ($\sum \left|\beta_{j}\right|$) forces some coefficients exactly to zero, enabling variable selection. The tuning parameter is determined using 3-fold cross-validation. More specifically, the tuning parameter λ was selected using Friedman’s “lambda.minimum” method to accurately quantify the independent contributions of climatic factors to rice yield and NUE [61].

Owing to the fact that each rice genotype exhibits distinct phenological stages (e.g., the period from sowing to heading), the climatic factors corresponding to its different growth stages also vary (see Supplementary Material Rar1 for details).The thresholds for high-temperature stress in this study were referenced from the research of Sánchez et al. [62], with the high-temperature thresholds set at maximum air temperature ≥ 40.1 °C (seedling stage), ≥35.3 °C (tillering stage), ≥33.1 °C (booting stage), ≥37 °C (flowering stage), and ≥31.3 °C (filling stage). The low-temperature thresholds were defined as a daily average air temperature < 12 °C at the seedling stage and <20 °C at other growth stages [63]. The flowering stage was defined as the period from when 80% of the plants in a plot started heading to when all plants reached full heading. Sunshine duration refers to the time during which the direct solar irradiance reaches or exceeds 120 W/m^2^. The weather data of Nanjing in this paper is derived from Lishui Meteorological Station of Jiangsu Province (station number: 58340), and that of Sanya is derived from Sanya Meteorological Station of Hainan Province (station number: 59948).

Data processing in this study was performed using SPSS 22.0 for correlation analysis and ANOVA. Pearson’s correlation method was used for correlation analysis, and differences in ANOVA were determined by Duncan’s multiple range test with a significance level of *p* < 0.05. Graphs and charts were generated using GraphPad 8.0.2, and the standard errors of the data presented in the paper are represented by SE values. Correlation heatmaps and Mantel test plots were generated using ChiPlot (https://www.chiplot.online/) (accessed on 31 May 2025).

## 5. Conclusions

Through LASSO regression, this study systematically deciphered the stage-specific effects of climatic factors, nitrogen application rate, and rice genotypes on yield, NUE, and nitrogen metabolism enzyme activities. Notably, these findings are site-, season- and genotype-specific within the tested environmental range, and the LASSO model identifies dominant associations rather than universal rules. This study provides a quantitative reference for understanding rice responses to climatic variation and weather stress. The core conclusions are as follows:

Rice exhibits significant growth stage-specificity in its response to climatic stress. High-temperature stress during the flowering stage and low-temperature stress during the filling stage are the main factors, while rainfall during the seedling stage and solar radiation during the tillering stage positively promote yield and NUE.

GSA and NRA during the filling stage show strong associative links between environmental stress and yield formation. Their explanatory power for yield (R^2^ > 0.75) is significantly higher than that of NPR, suggesting that nitrogen assimilation may represent an important limiting process for yield formation under climatic stress.

LASSO regression provides an effective framework for identifying genotype-specific response patterns under climatic variability. Among the tested materials, genotype 6 (YZ3) showed favorable yield and NUE responses under multiple environmental conditions, indicating its potential value as a candidate germplasm for stress-responsive breeding programs.

## Figures and Tables

**Table 1:** LASSO regression analysis of the associative effects of climatic factors, Nitrogen application, and rice genotypes on yield and NUE traits.

	Grain Yield	NHI	PNUE	NPFP	NRE
Intercept	10.4	0.586	31.4	68.8	0.700
Nitrogen application	0.0452		−0.0388	−0.0672	−0.00061
Days of high-temperature stress during tillering stage			−0.191	−0.424	
Days of high-temperature stress during booting stage				−0.0243	
Days of high-temperature stress during flowering stage	−0.164				−0.00142
Days of low-temperature stress during flowering stage				−1.01	−0.00557
Days of low-temperature stress during filling stage	−0.686			−0.465	−0.00796
Solar radiation during tillering stage	0.949	0.00125	0.716		0.0116
Solar radiation during booting stage				−0.401	
Solar radiation during filling stage		−0.00415		−0.17	
Sunshine duration during seedling stage	−3.95	−0.0511	−4.35	−4.75	−0.03175
Sunshine duration during flowering stage				−2.54	−0.00345
Rainfall during seedling stage	8.05	0.135	19.3	19.8	
Rainfall during booting stage	−0.135				
Rainfall during flowering stage	−0.0829		−0.0319		−0.00406
Rainfall during filling stage	−0.0212		−0.169	−0.439	
Genotype 1	−2.31			−4.52	−0.0581
Genotype 2		0.0664	14.1	1.66	−0.00416
Genotype 3	−2.26	−0.0139	−6.3	−8.21	
Genotype 4	2.87		4.15		0.0516
Genotype 5	1.16		4.48	9.16	0.148
Genotype 6	0.826	0.0193	3.09	1.13	
Genotype 7			−1.64	−0.0575	−0.0167
Genotype 8	−0.471	−0.0170	−3.22	−0.314	0.0081
Genotype 9		−0.0153			
lambda	0.341	0.01	0.499	0.366	0.00656
R^2^	0.875	0.663	0.808	0.799	0.535

Note: NHI: Nitrogen harvest index; PNUE: Physiological nitrogen use efficiency; NPFP: Nitrogen partial factor productivity; NRE: Nitrogen recovery efficiency. Empty cells indicate variables with regression coefficients shrunk to 0 and eliminated by LASSO regression. Columns denote response variables (Yield and NUE traits); Rows denote explanatory variables (Nitrogen application, climatic factors at different growth stages and rice genotypes).

**Table 2:** LASSO regression analysis of the associative effects of climatic factors, nitrogen application, and rice genotypes on NRA, GSA, and NPR during the booting and filling stages.

	NRA During Filling Stage	NRA During Booting Stage	GSA During Filling Stage	GSA During Booting Stage	NPR During Filling Stage	NPR During Booting Stage
Intercept	−14.5	−43.2	2.47	−5.94	12	10.0
Nitrogen application	0.178	0.0838	0.0325	0.0216	0.0330	0.00516
Minimum air temperature		0.480		0.189	1.08	0.141
Maximum air temperature	0.433	1.43	0.318	0.446	−1.51	0.262
Solar radiation					0.606	
Sunshine duration					1.33	0.190
Rainfall	4.18	0.663	0.372	0.107	0.857	
Genotype 1					−6.44	
Genotype 2		−4.55			−2.60	
Genotype 3					6.06	
Genotype 4						
Genotype 5		−2.23			0.420	
Genotype 6	13.4			1.54	−2.58	
Genotype 7					−2.09	
Genotype 8					−2.53	
Genotype 9					3.69	
lambda	7.22	0.937	1.10	0.600	0.00156	0.870
R^2^	0.469	0.817	0.563	0.777	0.845	0.457

Note: NRA: Nitrate reductase activity; NPR: Net photosynthetic rate; GSA: Glutamine synthetase activity. Empty cells indicate variables with regression coefficients shrunk to 0 and eliminated by LASSO regression. Columns denote response variables (NRA, GSA, NPR at the booting/filling stages); Rows denote explanatory variables (nitrogen application, climatic factors, and rice genotypes).

**Table 3:** Genotypes for breeding test.

Response Variable Name	Genotypes Name	Maternal Parent	Paternal Parent
Genotype 1	DFE02	/	/
Genotype 2	KASALATH	/	/
Genotype 3	YZ1	DFE02 (Japonica)	KASALATH (Indica)
Genotype 4	YZ2	DFE02 (Japonica)	KASALATH (Indica)
Genotype 5	IR30	/	/
Genotype 6	YZ3	DFE02 (Japonica)	IR30 (Indica)
Genotype 7	YZ4	DFE02 (Japonica)	IR30 (Indica)
Genotype 8	YZ5	DFE02 (Japonica)	IR30 (Indica)
Genotype 9	YZ6	DFE02 (Japonica)	IR30 (Indica)

## Data Availability

The data and code of this study have been provided in the Supplementary Materials.

## Supplementary Materials

The following supporting information can be downloaded at: https://www.mdpi.com/article/10.3390/plants15040639/s1, Figure S1: Daily solar radiation and sunshine duration during the entire rice growing season from 2022 to 2024.; Figure S2: Changes in fitting deviation of traits with different *λ* values. Figure S3. Yield of nine rice genotypes. Table S1: Correlation coefficients among nitrogen metabolism enzyme activities, NPR, and yield; Table S2: LASSO regression analysis of rice traits and climatic factors. Rar1: Data and code of this study.

## Abbreviations

The following abbreviations are used in this manuscript:

LASSO	Least absolute shrinkage and selection operator
NUE	Nitrogen use efficiency
NR	Nitrate reductase
GS	Glutamine synthetase
NRA	Nitrate reductase activity
GSA	Glutamine synthetase activity
NPR	Net photosynthetic rate
HI	Harvest index
NHI	Nitrogen harvest index
NPFP	Nitrogen partial factor productivity
NRE	Nitrogen recovery efficiency
PNUE	Physiological nitrogen use efficiency
LN	Low nitrogen
HN	High nitrogen
