# Supplementary material for: Stage-Specific Effects of Climatic Variation on Rice Yield and Nitrogen Use Efficiency for Identifying Adaptive Genotypes Based on Variable Selection Method
Source: Plants (Basel). 2026 Feb 18;15(4):639. doi: 10.3390/plants15040639 (PMC12944175; doi:10.3390/plants15040639)
Supplement: Supplementary file 1 [file plants-15-00639-s001.zip › plants-4079375-supplementary.pdf]

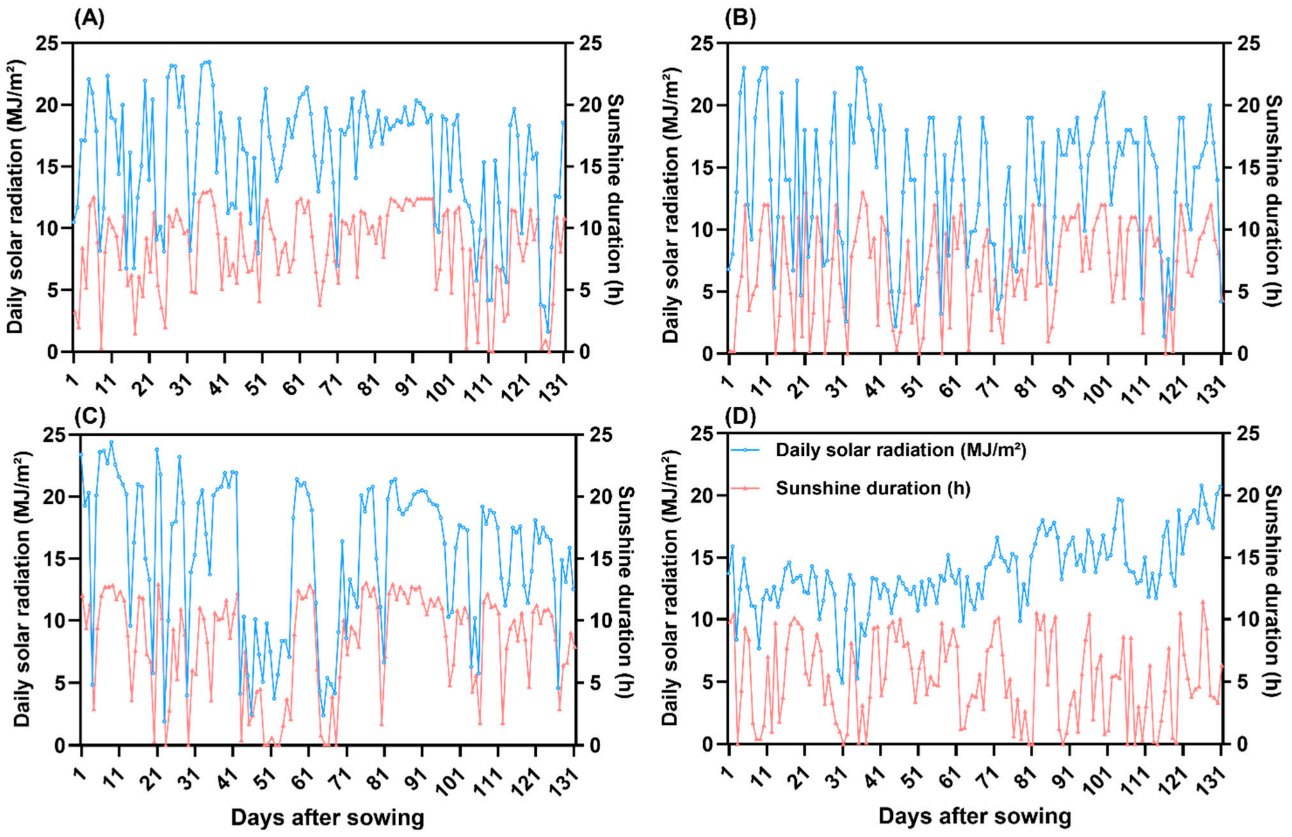

Figure S1. Daily solar radiation and sunshine duration during the entire rice growing season from 2022 to 2024. (A): 2022 Nanjing; (B): 2023 Nanjing; (C): 2024 Nanjing; (D): 2024 Sanya

Table S1. Correlation coefficients among nitrogen metabolism enzyme activities, NPR and yield

	GSA during filling stage	GSA during booting stage	NPR during filling stage	NPR during booting stage	NRA during filling stage	NRA during booting stage
Correlation coefficient with grain yield	0.7759***	0.4291***	0.0058	0.0014	0.7951***	0.5245***

Note: *** indicates significances at $p = 0.001$ levels.

Table S2. LASSO regression analysis of rice traits and climatic factors

	Straw Weight	Biomass	Nitrogen concentration in grain	Nitrogen concentration in straw	Nitrogen accumulation in grain	Nitrogen accumulation in straw	Harvest index	Nitrogen accumulation in plant
Intercept	−3.55	−45.7	12.5	11.0	145	−9.86	0.476	215
Nitrogen application	0.0708	0.150	0.00803	0.00604	0.659	0.868	−0.00002	1.55
Days of high temperature stress during tillering stage		−0.163			−3.40		−0.00109	
Days of high temperature stress during flowering stage		−0.485			−0.548			
Days of high temperature stress during filling stage		−0.154			−0.151			
Days of low temperature stress during tillering stage			0.0184			1.79		
Days of low temperature stress during filling stage		−1.11	0.0878		−8.55	−0.446		−11.7
Maximum temperature during filling stage			−0.0186					
Solar radiation during tillering stage	0.170	6.22	−0.0350	−0.264	9.62			7.81
Solar radiation during booting stage	1.25	0.0613				1.79		4.36
Solar radiation during filling stage		0.0588			−1.56		−0.00332	
Sunshine duration during seedling stage					−42.3	7.78	−0.0521	−32.6
Sunshine duration during tillering stage				−0.129				
Sunshine duration during flowering stage		−2.01						
Rainfall during seedling stage			−0.908		75.7		0.142	
Rainfall during tillering stage	−0.373					−5.02	0.00509	
Rainfall during booting stage		−0.0188			−3.38			
Rainfall during flowering stage	−0.0846	−0.881	0.0100	0.000497		−1.03		−2.67
Rainfall during filling stage		−0.0909		−0.00274			−0.00166	−1.05
Genotype 1		−3.79			−15.8	−7.84		−34.7
Genotype 2			−0.263	−1.31		−54.3	0.0495	−61.7
Genotype 3		−2.40			−14.5		−0.0257	
Genotype 4		5.33			46.6		0.00621	9.15
Genotype 5		6.99			35.8	32.7	0.0415	30.9
Genotype 6					20.9	−19.3	0.0263	
Genotype 7						−0.854	−0.00526	
Genotype 8				0.531		26.2	−0.00793	13.5

Genotype 9		2.39	−0.546	0.0818	−6.59			
lambda	1.55	0.413	0.221	0.198	2.24	4.41	0.00478	7.24
R^2	0.538	0.815	0.572	0.429	0.898	0.669	0.800	0.836

Note: Empty cells indicate variables with regression coefficients shrunk to 0 and eliminated by LASSO regression. Columns denote response variables; Rows denote explanatory variables (Nitrogen application, climatic factors at different growth stages and rice genotypes).

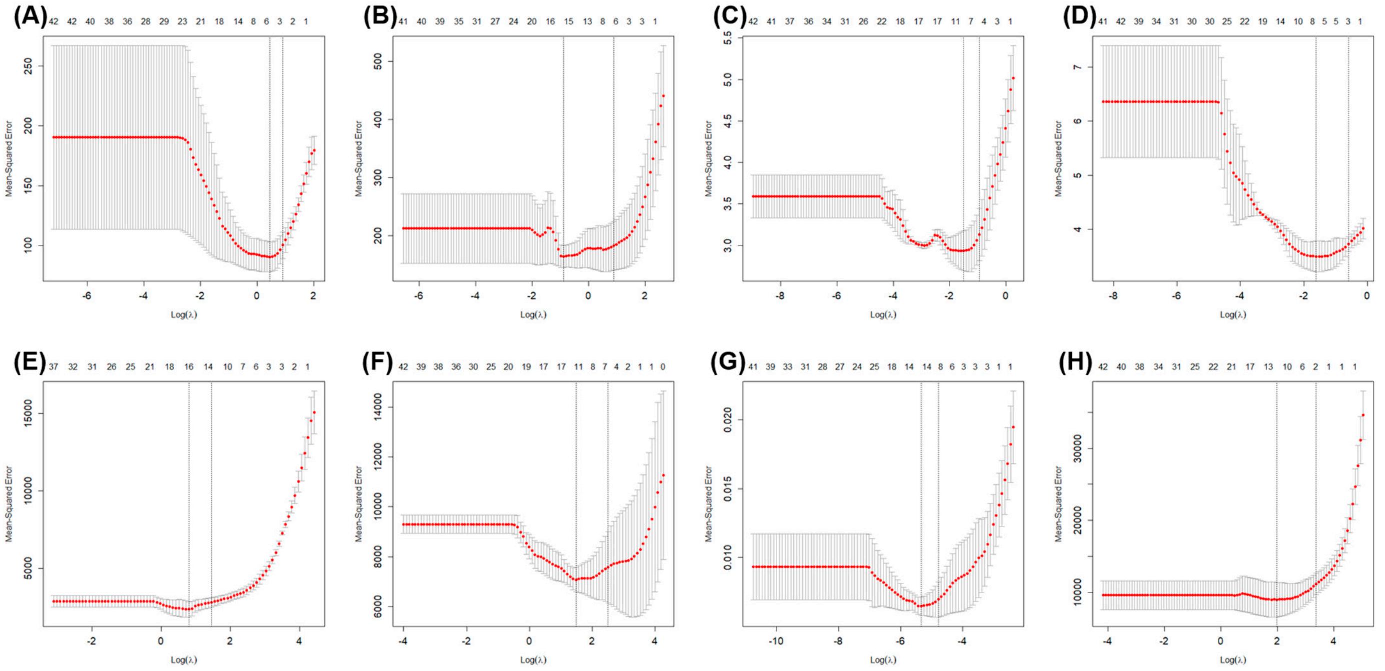

Figure S2. Changes in fitting deviation of traits with different λ values. (A): Straw weight; (B): Biomass; (C): Nitrogen concentration in grain; (D): Nitrogen concentration in straw; (E): Nitrogen accumulation in grain; (F): Nitrogen accumulation in straw; (G): Harvest index; (H): Nitrogen accumulation in plant. The left vertical dotted line represents lambda.min and the right vertical dotted line represents lambda.1se. The red dots represent the mean-squared error, and the error bars represent the standard deviation of the mean-squared error.

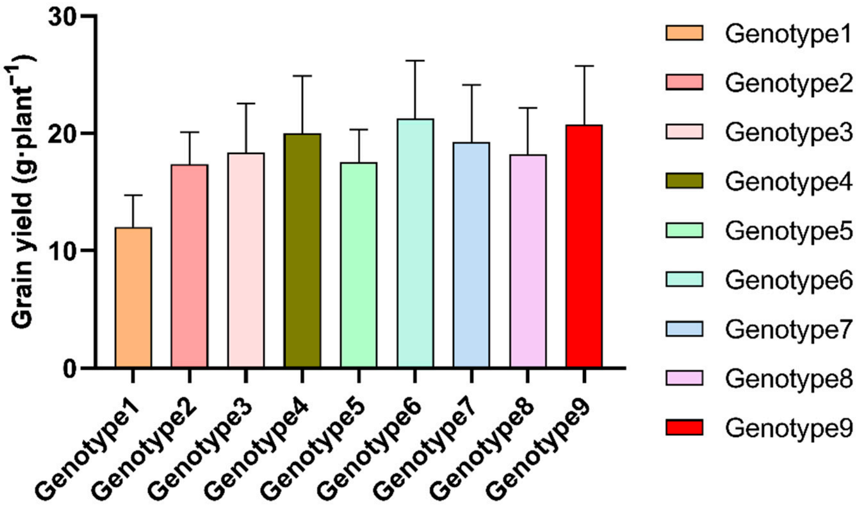

Figure S3. Yield of nine rice genotypes. Data are presented as mean ± SE. No significant difference was observed among genotypes ($p > 0.05$; Duncan's multiple range test).
